# The miRNA–mRNA Networks Involving Abnormal Energy and Hormone Metabolisms Restrict Tillering in a Wheat Mutant *dmc*

**DOI:** 10.3390/ijms20184586

**Published:** 2019-09-17

**Authors:** Junhang An, Hao Niu, Yongjing Ni, Yumei Jiang, Yongxing Zheng, Ruishi He, Junchang Li, Zhixin Jiao, Jing Zhang, Huijuan Li, Qiaoyun Li, Jishan Niu

**Affiliations:** 1 National Centre of Engineering and Technological Research for Wheat/Key Laboratory of Physiological Ecology and Genetic Improvement of Food Crops in Henan Province, Henan Agricultural University, Zhengzhou 450046, China; jhan68@163.com (J.A.); nxyjym@henau.edu.cn (Y.J.); 13253817312@stu.henau.edu.cn (Y.Z.); guomai301@163.com (R.H.); chang_top@163.com (J.L.); zxjiao2018@163.com (Z.J.); jzhang1023@126.com (J.Z.); lhj19960901@163.com (H.L.); liqiaoyun@henau.edu.cn (Q.L.); 2 Institute of Cotton Research, Chinese Academy of Agricultural Sciences, Anyang 455000, China; m15927306510@163.com; 3 Shangqiu Academy of Agricultural and Forestry Sciences, Shangqiu 476000, China; nyj317@163.com

**Keywords:** wheat (*Triticum aestivum* L.), *dmc* mutant, tillering, miRNA, mRNA, photosynthesis, carbohydrate, phytohormone

## Abstract

Tillers not only determine plant architecture but also influence crop yield. To explore the miRNA regulatory network restraining tiller development in a dwarf-monoculm wheat mutant (*dmc*) derived from Guomai 301 (wild type, WT), we employed miRNome and transcriptome integrative analysis, real-time qRT-PCR, histochemistry, and determinations of the key metabolites and photosynthesis parameters. A total of 91 differentially expressed miRNAs (DEMs) were identified between *dmc* and WT. Among them, 40 key DEMs targeted 45 differentially expressed genes (DEGs) including the key DEGs encode growth-regulating factors (GRF), auxin response factors (ARF), and other proteins involved in the metabolisms of hormones and carbohydrates, etc. Compared with WT, both the chlorophyll contents and the photosynthesis rate were lower in *dmc*. The contents of glucose, sucrose, fructose, and maltose were lower in *dmc*. The contents of auxin (IAA) and zeatin (ZA) were significantly lower, but gibberellin (GA) was significantly higher in the tiller tissues of *dmc*. This research demonstrated that the DEMs regulating hormone and carbohydrate metabolisms were important causes for *dmc* to not tiller. A primary miRNA–mRNA regulatory model for *dmc* tillering was established. The lower photosynthesis rate, insufficient energy, and abnormal hormone metabolisms restrict tillering in *dmc*.

## 1. Introduction

Tillers are special vegetative branches at the base of monocot plants. They are developed from the axillary meristems at the first several nodes of the monocot main culm, and their shape and height are similar to the main culm [1,2]. Tillering ability is one of the most important agricultural traits which significantly affect grain yield. Common wheat (*Triticum aestivum* L.) is one of the most valuable and widely planted monocot plants. Increasing productive tiller number generally enhances yield potential. However, wheat cultivars with too few or too many tillers do not have very high grain yield. Thus, optimal tiller number has always been the need for agricultural production [2,3].

Mutants with various tillering abilities are ideal materials for the studies of tiller developmental molecular mechanisms. Many rice (*Oryza sativa*) tiller mutants have been reported, such as *moc1*, which encodes a protein which can interact with MOC1 interacting protein and is the first characterized key gene controlling rice tillering [4,5]. Rice *nal2 nal3* (*nal2/3*) double recessive mutant encodes WUSCHEL-relate homeobox 3A (OsWOX3A), which is involved in formation of tillers [6]. Several wheat tiller inhibition lines or mutants have been reported; they harbor genes *tin1* [7], *tin2* [8], *tin3* [9], and *ftin* [10]. One wheat high tillering mutant NAUH167 harbors a major quantitative trait locus (QTLs) [11]. Wheat *QSR.sicau-4D* may be a pleiotropic QTL controlling maximum tiller number per unit area [3]. Although there are some reports about wheat tillering, the molecular mechanisms of wheat tillering are largely unknown. The regulatory signals, genes, and miRNAs involved in wheat tillering need to be investigated.

MiRNAs are ~22 nucleotide (nt)-long non-coding RNAs playing essential regulatory roles in eukaryotic genomes. They regulate gene expressions by silencing their target genes at the posttranscriptional level [12,13,14]. The first set of plant miRNAs were reported in *Arabidopsis thaliana* in 2002 [15,16]. In recent years, a number of plant miRNAs (18–25 nt) and their target genes have been reported to regulate various developmental processes, including branching or tillering [17,18,19]. For example, squamosa promoter binding protein-like genes (*SPLs*) were predicted as targets of miR156. Overexpressing miR156 inhibits the tiller or branch development in different plants, such as maize (*Zea mays*) [20], rice [21], Arabidopsis [22], and wheat [23]. In Arabidopsis, miR172 acts downstream of miR156, which regulates the expression of miR172 via SPL9 and promotes the juvenile-to-adult transition [24]. The expression of transcription factor (TF) RAP2.6L affects shoot apical meristems in Arabidopsis. RAP2.6L is under direct transcriptional control of miRNA-regulated class III homeodomain-leucine zipper (HD-ZIP III) proteins, key regulators of shoot meristem development [19]. In rice, the overexpression of *OsmiR393* increases its tillering ability [17]. Rice plants overexpressing osa-miR171b have thicker tillers, but their number of tillers is the same as control [25]. Overexpression of miR156 causes lower miR172 expression in a maize *Corngrass1* (*Cg1*) mutant, which is a neotenic mutation [20]. MiR167 determines the plant developmental process by regulating the expression of certain auxin response factor (*ARF*) genes involved in the auxin signaling pathway. Rice overexpressing miR167 decreases the expression of *OsARF*, and reduces tiller number [26]. Rice miR164b modulates the expression of *OsNAC2* and affects its stem development [27]. In switchgrass (*Panicum virgatum*), overexpressing miR393 raises tillering capacity through regulation of auxin signaling transduction [28]. Although there is some literature on miRNA regulating plant branching, how miRNA networks regulate tillering in monocots is largely unknown.

Most miRNAs and their functions are conserved in various plant species, but some of them are divergent. Some families—such as miR165/6, miR156/7, miR159, miR170/1, miR319, miR390, miR396, etc.—are present in all vascular plants studied, but miR6300 and miR5077 may be conserved only in lycophytes and ferns, and miR168 and miR172 are not found in lycophytes [29]. In common wheat, a number of miRNAs have been reported [30,31,32,33,34,35], but their functions still need to be carefully investigated. Although wheat is a widely planted crop, how miRNA regulates its tillering is almost unknown. Up to now, we only know that miR156-Squamosa Promoter Binding Protein-Like (TaSPLs) and strigolactone (SL) signaling pathways may modulate bread wheat tillering [23].

Previously, we reported a dwarf-monoculm wheat mutant (*dmc)* mutant, which was obtained from ethylmethane sulfonate (EMS) treated winter wheat cultivar Guomai 301. The mutant, *dmc,* almost doesn’t tiller in certain field conditions. The down-regulated gene expressions related to phytohormone syntheses of auxin (IAA), zeatin (ZA), cytokinin (CTK), and some TF families of TALE and WOX are the major causes of the mutant *dmc* not tillering [36]. The mutant, *dmc,* provided an opportunity to design experiments to determine the miRNA networks regulating wheat tillering. Here we report the miRNA–mRNA networks regulating wheat tillering in *dmc*.

## 2. Results

### 2.1. The Tiller Primordia of dmc Cannot Grow Out

**Figure 1:**
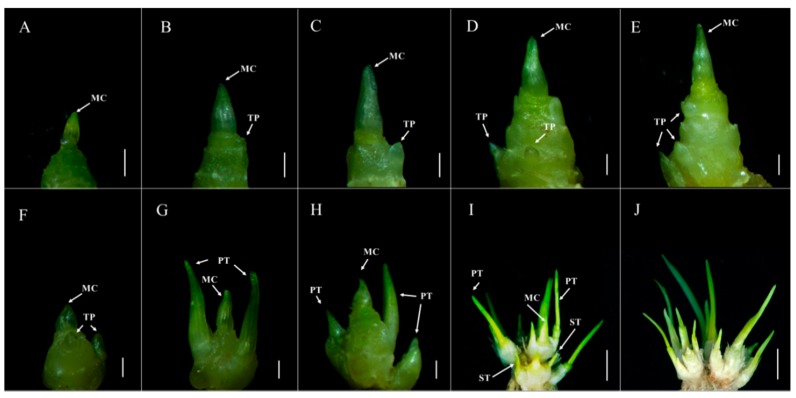
The tiller micromorphology of dwarf-monoculm wheat mutant (*dmc)* (**A**–**E**) and WT (**F**–**J**). A, B, C, D, E: *dmc* at the two-leaf stage, three-leaf stage, four-leaf stage, over-winter stage, and between the rising and jointing stage. F, G, H, I: WT at the two-leaf stage, three-leaf stage, four-leaf stage, and over-winter stage. J: some tillers of WT at between the rising and jointing stage. MC: main culm; TP: tiller primordium; PT: primary tiller; ST: secondary tiller. A-H, scale bar = 1 mm; I and J, scale bar = 1 cm.

The tiller morphology and microstructure of *dmc* and wild type (WT) were compared (Figure 1, Figure S1). In *dmc*, the tiller differentiation and development at the main culm (MC) base were severely inhibited (Figure 1A–E). At the two-leaf stage, there almost no difference between *dmc* and WT outside; however, the tiller primordia (TP) of WT already began to differentiate (Figure 1F), but no TP could be observed on *dmc* (Figure 1A). At the three-leaf stage, the tiller buds of WT began to grow and formed two primary tillers (PT) at the base of MC (Figure 1G); meanwhile, only one tiny protuberance formed at the MC base of *dmc* (Figure 1B). At the four-leaf stage, the MC of WT grew thick and long and grew more PTs (Figure 1H). Meanwhile, the MC of *dmc* also elongated, and one tiny TP at the base could be observed (Figure 1C). At the over-winter stage, the average plant height of the *dmc* was only 12 cm (the highest leaf to the ground in the natural state), which was half of the WT (Figure S1B). There were only two tiny TPs at the base of the *dmc* (Figure 1D), but WT was at the tiller exuberance stage; the number of tillers was about 12–14, which mainly consisted of PT and ST (Figure 1I). Between the rising to the jointing stage, the tiny TPs of *dmc* were very small, as before (Figure 1E); however, the tiller number of WT had reached its maximum (Figure 1J). Most of the *dmc* had only one MC in the end (Figure S1C).

### 2.2. The Protein Content of dmc Tiller Primordia Was Less

**Figure 2:**
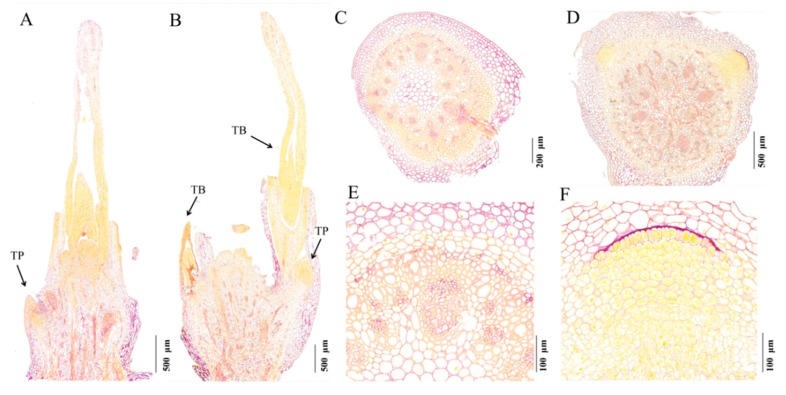
The distribution of starch (purple) and protein (yellow) in wheat tillers of WT and *dmc* at the three-leaf stage. Tiller histological section micrographs of WT (**B**,**D**,**F**) and mutant *dmc* (**A**,**C**,**E**). (**A**) Longitudinal section of the tiller bud of *dmc* (arrowhead); (**B**) Longitudinal section of tillers of WT (arrowheads); (**C**) Transection of the tiller base of *dmc*; (**D**) Transection of the tiller base of WT; (**E**) An enlarged view of C; (**F**) An enlarged view of D. TP: tiller primordium; TB: tiller bud.

At the histological level, the tiller primordia of *dmc* were distinguishable from that of the WT at the three-leaf stage. The *dmc* usually had only one small tiller protuberance, which was stained very faint yellowish purple (Figure 2A); in contrast, the WT usually had two tillers, which were stained deep yellow (Figure 2B). These phenotypes indicated that the protein content in the tiller buds of WT were significantly more than that in *dmc*. Additionally, in the transverse sections of WT, there were two distinct yellow regions (Figure 2D), which were the primordia of exuberant tillers. However, there usually no tiller primordium could be observed in *dmc* (Figure 2C). The cells of the primordia were arranged orderly and closely, the cytoplasm was density and stained deep yellow in WT (Figure 2F) and exhibited the characteristics of the typical meristematic tissues. Contrarily, almost no tiny primordia meristematic tissues could be seen in *dmc* (Figure 2E). In summary, *dmc* had few tillers, and their tiller primordia had less protein than that of the WT.

### 2.3. Overview of the miRNome Data

We obtained a total of 115,402,489 raw reads, 53,485,942 and 61,913,378 clean reads with lengths of 18–30 bp from WT and *dmc* (Table S1). The rRNA, tRNA, snRNA, snoRNA, and repeat sequences were removed from the clean reads, and we obtained a total of 77,776,843 reads (Table S2). There were 47,283,166 (60.79%) reads which were mapped to the wheat reference genome. A total of 454 miRNAs belonging to 84 families were obtained from the two samples (Table S3)—among them, 78 were known miRNAs, and 376 were novel miRNAs (Table S4). The most miRNAs were 21–24 nt in the six miRNA libraries (Figure S2).

### 2.4. DEMs between WT and dmc

**Figure 3:**
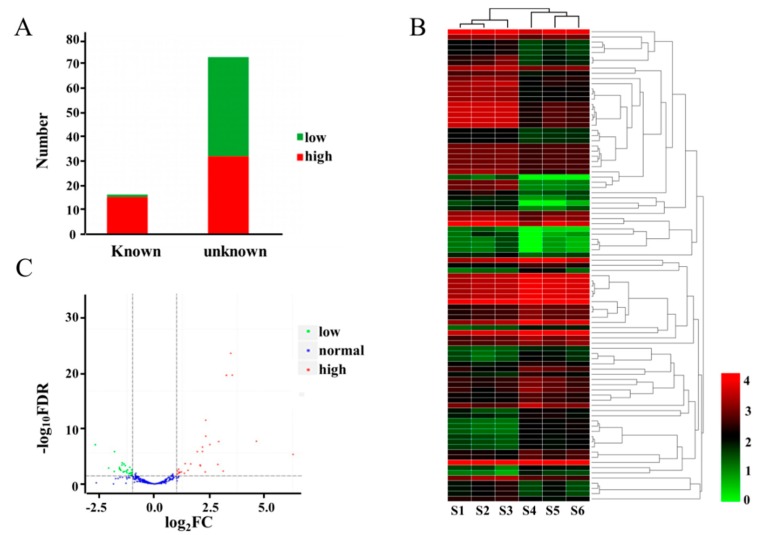
Expression profiles of the differentially expressed miRNAs (DEMs) between *dmc* and WT. (**A**) Stacking bar diagram of the DEMs; (**B**) Heatmap of the DEMs. S1, S2, S3: mutant *dmc*; S4, S5, S6: WT. The color scale indicates the values of LgFPKM; FPKM: fragments per kilobase of transcript per million. (**C**) Volcano plots of the DEMs. The red dots represent the highly expressed DEMs in *dmc*, green dots represent the lowly expressed DEMs in *dmc,* and blue dots represent no difference in DEM expression between *dmc* and WT; FC: fold change; FDR; false discovery rate.

A total of 91 miRNAs (*p* < 0.05) were significantly differentially expressed between *dmc* and WT (Figure 3, Table S5). Among the 91 differentially expressed miRNAs (DEMs), 17 of the miRNAs were known, while 74 were novel, of which 49 were highly expressed, and 42 were lowly expressed in *dmc* compared to WT (Figure 3A, 3C). The expression levels of DEMs were also hierarchically clustered (Figure 3B). The top three miRNAs with the biggest expression difference were all highly expressed in *dmc* (FC > 60). Among the 17 known miRNAs, tae-mir9655-3p, tae-miR399, and tae-miR9675-3p were the top three highly expressed miRNAs in *dmc* (FC > 10). tae-miR156 was highly expressed in *dmc* (FC > 4) with more than 2900 Transcripts Per Million (TPM), and, of the known miRNAs, had the highest abundance. Among the 74 novel miRNAs, five miRNAs, novel-10992, novel-4704, novel-13869, novel-22124, and novel-20106, were highly expressed in *dmc* (FC > 10), whilst novel-22869, novel-393 and novel-4827 were highly expressed in WT (FC > 6). Novel-48136 was also highly expressed in WT (FC > 3) with more than 7000 TPM, and was the highest abundance novel miRNA, which suggested that it might be the major miRNA related to wheat tillering. These significant DEMs were the key regulatory miRNAs causing the phenotype of *dmc*.

### 2.5. The DEMs’ Targets and Their Functions

**Figure 4:**
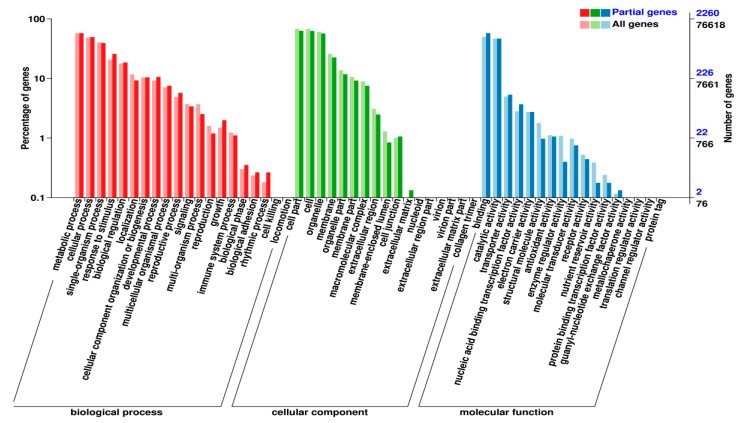
Functional classification of the target genes of the DEMs, referring to the GO database.

To gain insight into the functions of the DEMs during wheat tillering, we predicted their potential target genes and annotated them, referring to the database Gene Ontology (GO). Functional classification showed that the following aspects were significantly different between mutant *dmc* and WT: (1) biological process: multi-organism process and rhythmic process; (2) cellular component: membrane-enclosed lumen and extracellular region; (3) biological process: structural molecule activity, enzyme regulator activity, and nutrient reservoir activity, which were closely related to the phenotype of *dmc* (Figure 4).

A total of 3254 mRNAs were targeted by the 91 DEMs, and 299 GO terms were selected at *P* < 0.05 (Table S6). The top three most enriched GO terms were the apoptotic process (GO:0006915), defense response (GO:0006952), and adenosine diphosphate (ADP) binding (GO:0043531).

The Kyoto Encyclopedia of Genes and Genomes (KEGG) annotation indicated that the DEMs and their targets belonged to 69 KEGG pathways. The enriched pathways (Table 1) were protein processing in the endoplasmic reticulum (ko04141), spliceosome (ko03040), and circadian rhythm-plant (ko04712), etc. By comparing the KEGG pathways of DEMs with the KEGG pathways of differentially expressed genes (DEGs) derived from the same samples [36], nine the same KEGG pathways were found, including arginine and proline metabolism (ko00330), butanoate metabolism (ko00650), carbon metabolism (ko01200), carotenoid biosynthesis (ko00906), fatty acid elongation (ko00062), fructose and mannose metabolism (ko00051), glycerophospholipid metabolism (ko00564), phenylpropanoid biosynthesis (ko00940), and starch and sucrose metabolism (ko00500), which were predicted to be related to tillering.

### 2.6. The Photosynthesis of dmc Was Weak at Tillering Stage

**Figure 5:**
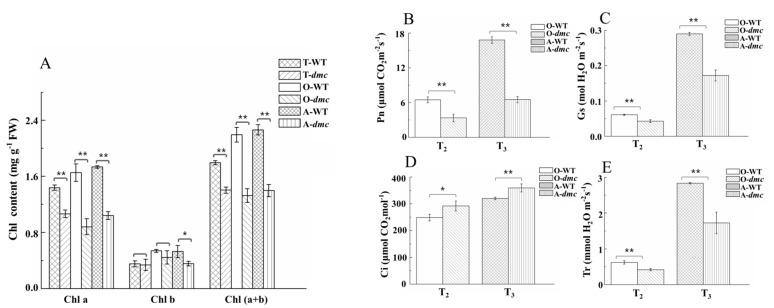
The chlorophyII contents and photosynthetic parameters of the fully expanded main-stem leaves of *dmc* and WT during tillering. **(A**) Leaf chlorophyII content. Chl a, the content of chlorophyII a; Chl b, the content of chlorophyII b; Chl (a + b), the content of chlorophy II a + b. (**B**–**E**) Leaf photosynthesis parameters. Each bar represents the mean ± SD of three biological replicates. Asterisks indicate a statistically significant difference between *dmc* and WT at the same stage (*: *P* ≤ 0.05, **: *P* ≤ 0.01). T-WT: WT at the three-leaf stage; T-*dmc*: *dmc* at the three-leaf stage; O-WT: WT at the over-winter stage; O-*dmc*: *dmc* at the over-winter stage; A-WT: WT between the rising to jointing stage; A-*dmc*: *dmc* between the rising to jointing stage.

The leaf contents of chlorophyll a (Chl a), chlorophyll b (Chl b), chlorophyll (a + b) (Chl (a+b)) were significantly lower in *dmc* than that in WT at the three-leaf stage (T_1_), over-winter stage (T_2_), and the rising to jointing stage (T_3_) except the Chl b at T_1_ and T_2_ stages (Figure 5A, Table S7). The most significant difference was from Chl a. In *dmc*, the Chl a of the fully unfolded new leaves at the T_2_ and T_3_ stages had decreased by 17% and 2% compared to that at T_1_ stage. However, the Chl a of WT continuously increased during tillering, and they were 1.35, 1.88, and 1.67-fold of those in *dmc* at the same three stages. The contents of Chl b at the T_3_ stage and Chl (a + b) were also significantly different between *dmc* and WT, similar to that of Chl a (*P* ≤ 0.05).

Under natural conditions, the net photosynthetic rate (Pn), stomatal conductance (Gs), and transpiration rate (Tr) of *dmc* were significantly lower than that of WT at the T_2_ and T_3_ stages (Figure 5B–E, Table S7). The most significant difference was Pn. The average Pn value of WT was more than two times those of *dmc* at the T_3_ stage. In opposite, the internal CO_2_ concentration (Ci) was higher in *dmc* than that in WT, whether at the T_2_ or T_3_ stage. The Pn and Gs increased by about 49% and 75% in *dmc,* and 61% and 79% in WT; Ci and Tr increased by about 18% and 76% in *dmc,* and 22% and 78% in WT from the T_2_ to T_3_ stage. All the change rates of WT were higher than that of *dmc*. Obviously, the photosynthesis capacity of *dmc* was significantly lower than that of WT during tillering; this was an important cause for the lack of energy in *dmc*, which in turn affected its tillering.

### 2.7. Carbohydrate Contents in Leaves and Tiller Nodes of dmc and WT During Tillering

**Figure 6:**
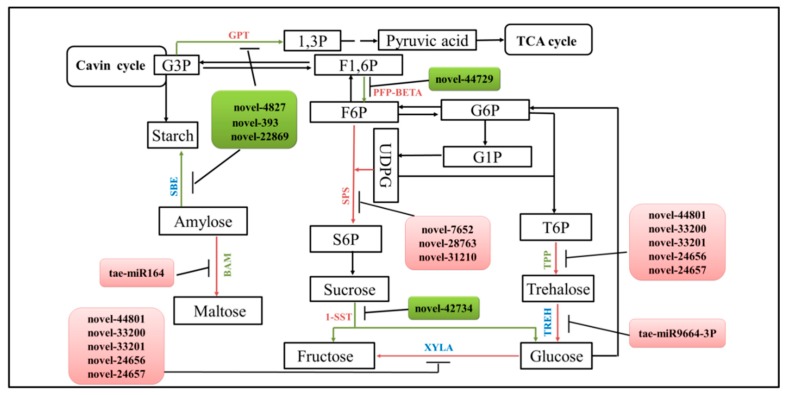
The key carbohydrate metabolic pathway and the miRNA–mRNA interactions in *dmc.* Pink: highly expressed in *dmc*; green: lowly expressed in *dmc*; blue: no difference in expression between *dmc* and WT. G3P: glycerol-3-phosphate transporter 1 (At3g47420); SBE: 1,4-alpha-glucan-branching enzyme (SBE1); Beta-amylase (BAM); PEP-BETA: pyrophosphate-fructose 6-phosphate 1-phosphotransferase subunit alpha (PEP-BETA); SPS: sucrose-phosphate synthase 4/5 (SPS4/SPS5); 1-SST: sucrose 1-fructosyltransferase (1-SST); XYLA: xylose isomerase (XYLA); TPP: trehalose-phosphate phosphatase 2 (TPP2); TREH: trehalase (TREH); F6P: fructose-6 -phosphate; G6P: glucose-6-phophate; F1,6P: 1,6-fructose diphosphate; S6P: sucrose-6-posphate; T6P: trehalose-6-phosphate; G1P: glucose-1-phosphate; 1,3P: 1,3-diphosphoglycerate; UDPG: uridine diphosphate glucose.

Transcriptomic analysis indicated that the starch and sucrose metabolism (ko00500) was one of the top ten enhanced KEGG pathways. In this study, fourteen significant DEMs were found to target DEGs involved in carbohydrate metabolism (Figure 6). Among them, novel-44801, novel-33200, novel-33201, novel-24656, and novel-24657 were highly expressed in *dmc*; they regulate trehalose and fructose synthesis. miR9664-3P was highly expressed in *dmc* and regulates glucose synthesis; novel-42734 was lowly expressed in *dmc* and regulates sucrose hydrolysis; miR164 was highly expressed in *dmc* and regulates polysaccharide hydrolysis, and maltose synthesis; novel-4827, novel-393, and novel-22869 were lowly expressed in *dmc* and regulate starch biosynthesis.

**Figure 7:**
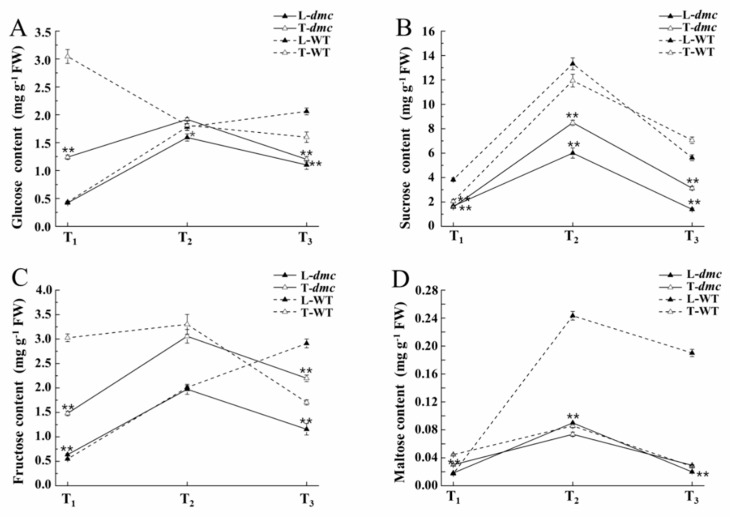
The carbohydrate contents in leaves and tiller nodes of *dmc* and WT. (**A**) Glucose content; (**B**) Sucrose content; (**C**) Fructose content; (**D**) Maltose content; L-WT: WT leaves; T-WT: WT tiller nodes; L-*dmc*: *dmc* leaves; T-*dmc*: *dmc* tiller nodes; T_1_: three-leaf stage; T_2_: over-winter stage; T_3_: between the rising to jointing stage (*: *P* ≤ 0.05, **: *P* ≤ 0.01).

In order to confirm the photosynthesis and carbohydrate metabolic results from the mRNA and miRNA analyses, the contents of four soluble sugars—glucose, sucrose, fructose and maltose—in leaves and tiller nodes of *dmc* and WT were determined (Figure 7; Table S8). Generally, the soluble sugar contents in tiller nodes of *dmc* were significantly lower. The glucose contents in the leaves and tiller nodes of *dmc* initially increased, then gradually decreased (Figure 7A). In contrast, the glucose contents in the leaves of WT increased gradually, but the glucose contents in the tiller nodes decreased gradually during tillering. The difference of glucose in tiller nodes at the T_1_ stage was the biggest; the glucose contents of WT were almost three times as that of *dmc*. Sucrose was the predominant soluble sugar in both *dmc* and WT (Figure 7B). All the contents were initially increased and then decreased in both leaves and tiller nodes. The sucrose contents in leaves and tiller nodes of *dmc* were less than those of the WT at the three stages. The decreased sucrose contents of *dmc* may be related to its low photosynthetic capacity and the decreased sucrose transport from leaves to tiller nodes. The fructose contents in the leaves of *dmc—*and tiller nodes both of *dmc* and WT—initially increased (Figure 7C), and then gradually decreased, but increased continuously in leaves of WT. At the T_1_ stage, fructose in tiller nodes of *dmc* was less than that in WT, but at the T_2_ stage, there was almost no difference between *dmc* and WT. At the T_3_ stage, the contents of fructose in tiller nodes of *dmc* were more than those in WT. Maltose contents were the least among the four determined soluble sugars (Figure 7D). At the T_1_ stage, there was no difference in maltose contents in leaves of *dmc* and WT, but the maltose contents in tiller nodes of *dmc* were less than those of WT. At the T_2_ and T_3_ stages, there was no difference in maltose contents in tiller nodes, but maltose contents in leaves of *dmc* were lower than those of WT.

### 2.8. The IAA, ZA, GA Metabolism Pathways in dmc

**Figure 8:**
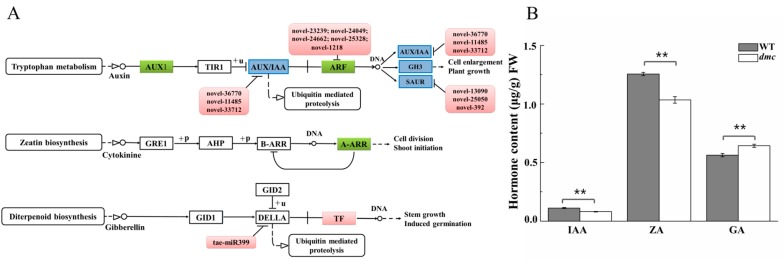
The auxin (IAA), zeatin (ZA) and gibberellin (GA) contents and the metabolism pathways. (**A**)The phytohormone metabolism pathways referenced to ko04075 in the KEGG database. (**B**) The contents of IAA, ZA, and GA in tiller nodes of *dmc* and WT. Pink: highly expressed in *dmc*; green: lowly expressed in *dmc*; blue: no difference in expression between *dmc* and WT (**: *P* ≤ 0.01).

Transcriptomic analysis indicated that there were 11 DEGs—*IAA14*, *IAA31*, *IAA2*, *IAA27*, *IAA25*, *ARF11*, *ARF2*, *GH3.7*, *NPF2.3*, *GH3.11*, and *ARG7*—involved in IAA metabolism; one down-regulated gene, *ARR9*, involved in ZA metabolism, and one up-regulated gene, *PIF3,* involved in GA metabolism (Figure S3) [36]. Based on the KEGG analysis of the miRNA target genes and the expression of miRNAs, the eleven miRNAs were all highly expressed in auxin metabolisms, and miR399 was highly expressed in gibberellin biosynthesis in *dmc*. No DEMs regulating cytokinin metabolism were found (Figure 8A). Integrative analysis of miRNA and mRNA interaction found that *ARF* was down-regulated by five novel miRNAs: novel-23239, novel-24049, novel-24662, novel-25328, and novel-1218. The gibberellin 20 oxidase 2 (*20ox2*) gene was down-regulated by tae-miR164 (Table S9).

By comparing the hormone contents of *dmc* and WT at the three-leaf stage (Figure 8B, Table S10), we found that IAA and ZA were significantly less in *dmc* than that in WT, while GA were significantly more in *dmc* than that in WT. Among them, IAA in WT was 1.4-fold of that in *dmc*, ZA in WT was 1.2-fold of that in *dmc*, and GA in *dmc* was 1.1-fold of that in WT. These results were consistent with the sequencing results.

### 2.9. The miRNA–mRNA Networks Restricting dmc Tillering

**Figure 9:**
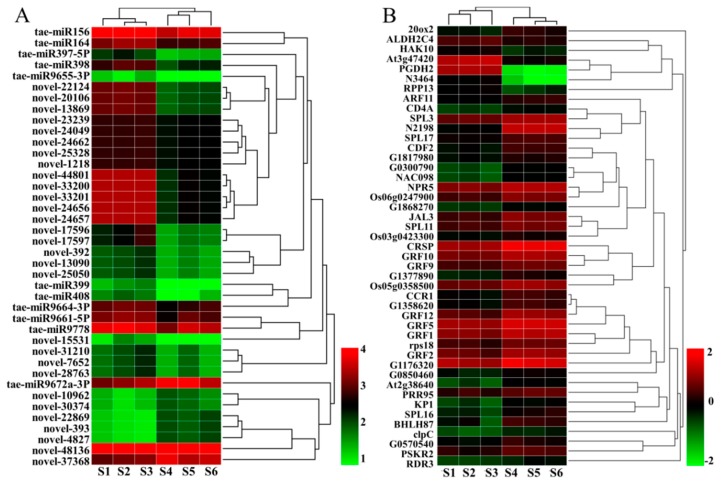
Heat maps of the differentially expressed miRNAs and mRNA between *dmc* and WT. (**A**) Heat map of the DEMs. (**B**) Heat map of the DEGs. S1, S2, S3: mutant *dmc*; S4, S5, S6: WT. The color scales indicate the values of LgFPKM.

**Figure 10:**
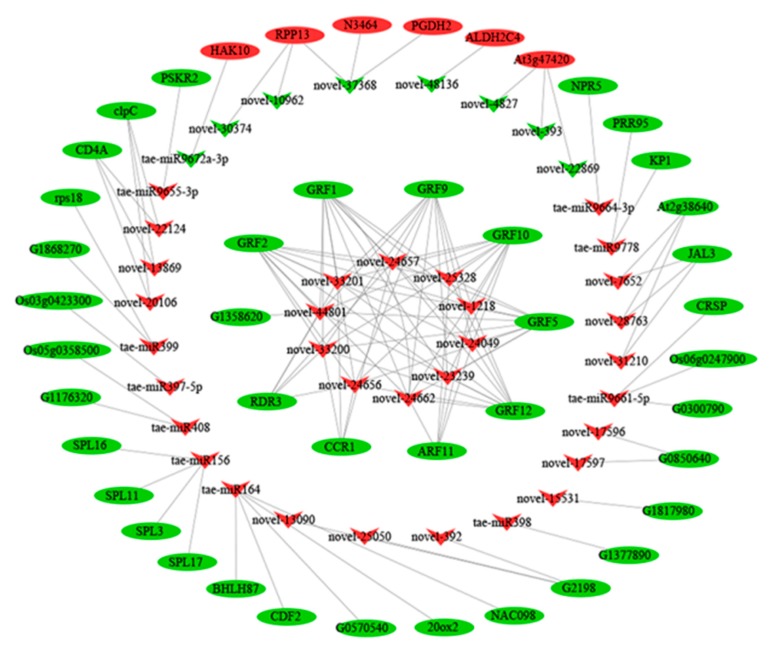
Negative regulation networks between the key DEMs and their target DEGs in *dmc.* The red indicates highly expressed in *dmc*, the green indicates lowly expressed in *dmc*, ellipses indicate DEGs, arrowheads indicate DEMs, the lines indicate the regulation relationship.

The transcriptome and miRNome data were integrated and analyzed to find out the miRNA–mRNA networks restricting *dmc* tillering. Only the pairs of DEM–DEG with inverse expression profiles were selected for further functional analyses. Some DEMs only had one predicted target gene, but most DEMs had many target genes. Some target genes of the DEMs were also significant DEGs; the results here confirm that which are previously reported [36]. The data were shown in Table S11. In the end, 125 pairs of negative miRNA–mRNA interactions, including 40 DEMs, were identified. Thirty-four DEGs were known genes according to swiss-prot annotation. The expression patterns of DEMs and DEGs were hierarchically clustered (Figure 9). The regulation networks between the key DEMs and their target DEGs were displayed by Cytoscape (Figure 10, Figure S4, Table S9). Among the 40 miRNAs, 11 were known miRNAs, and 29 were novel miRNAs. One-third of the novel miRNAs belonged to the miR396 family, which mainly regulated GRF and ARF families and formed 65 pairs of miRNA–mRNA interactions. The novel-22869, novel-393, novel-4827, and tae-miR164 involved in carbohydrate and GA metabolism, respectively, were found.

### 2.10. Expression Profiles of Ten DEMs and Their Eight Target DEGs

**Figure 11:**
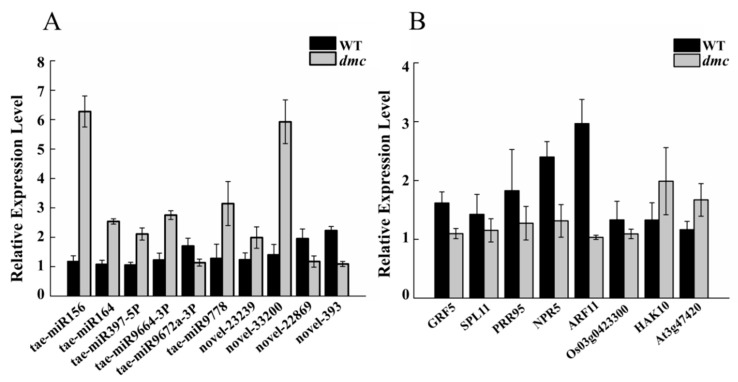
The expression profiles of ten DEMs and eight of their target DEGs. (**A**) The expression profiles of ten DEMs; (**B**) The expression profiles of eight target DEGs. *GRF5* was targeted by novel-23239, novel-33200; *SPL11* was targeted by tae-miR156; *PRR95* was targeted by tae-miR9778; *NPR5* was targeted by tae-miR9664-3P; *ARF11* was targeted by novel-23239; *Os03g0423300* was targeted by tae-miR397-5P; *HAK10* was targeted by tae-miR9672a-3P; *At3g47420* was targeted by novel-22869 and novel-393.

To confirm the reliability of the results derived from high-throughput miRNA sequencing and the miRNA–mRNA interaction analysis, we selected ten important DEMs and eight of their target genes to do real-time PCR (Figure 11A, Figure 11B, Table S9). Among them, six known miRNAs—miR156, miR164, miR397-5P, miR-9664-3p, and miR9778—and two novel miRNAs—novel-23239 and novel-33200—were highly expressed in *dmc*. Conversely, the novel-22869, novel-393, and miR-9672a-3p were lowly expressed in *dmc*.

The results indicated that all the target genes—including *GRF5* (growth-regulating factor), *SPL11* (squamosa promoter-binding-like protein), *HAK10* (potassium transporter 10), *PRR95* (two-component response regulator-like protein), *NPR5* (regulatory protein), *ARF11* (auxin response factor 11), Os03g0423300 (Acyl-[acyl-carrier-protein] desaturase 4), and At3g47420 (putative glycerol-3-phosphate transporter 1)—showed reverse expression profiles compared to their regulating miRNAs.

## 3. Discussion

### 3.1. The Tiller Primordia of dmc Lack Protein and Energy

Both wheat and rice tillers require exuberant meristems. Rice MOC1 acts to regulate the rice axillary meristem formation process [37]. In *dmc*, the tiller differentiation and development were severely inhibited. The chlorophyll contents and photosynthesis capacity of *dmc* plants were significantly lower (Figure 5). The tiller primordia had less protein content (Figure 2), less carbohydrate content (Figure 7), and lacked the viability of typical meristematic tissues (Figure 2). These factors led to a lack of enough energy and structural materials and caused the inability of the tiller primordia of *dmc* to grow out (Figure 1). 

### 3.2. The Important miRNAs Regulate Wheat Tillering

A total of 91 important DEMs were identified in tiller primordia of *dmc* and WT, including 17 known and 74 novel miRNAs, which enriched wheat miRNA data. The predicted target genes of the DEMs, such as structural molecule activity, enzyme regulator activity, and nutrient reservoir activity, were significantly lowly expressed in *dmc*, which were consistent with its dwarf phenotype and almost no tillers. Similarly, other predicted target genes were involved in several important metabolic pathways such as arginine and proline metabolism, carbon metabolism, and starch and sucrose metabolism, etc.

Ten key known and thirty novel miRNAs were found to play important roles in tiller development in miRNA regulation network through comparison of the *dmc* and WT transcriptomes and miRNomes (Figure 10, Figure S4, Table S9). The tae-miR156, tae-miR9778, and novel-33200 genes were highly expressed in *dmc.* According to previous reports, miR156 mainly inhibits plant tillering or branching in many species such as maize, rice, Arabidopsis, and wheat [20,21,22,23]. For example, rice miR156 negatively targets *OsSPL14*, and the expression level of *OsSPL14* and degree of tiller outgrowth suppression is clearly correlated [38]. These miRNAs reported here played important roles during wheat tillering.

### 3.3. The miRNAs Regulate Energy Metabolism in dmc

Previously we found that photosynthesis (ko00195) was the third most enhanced pathway in *dmc,* and carbohydrate-related metabolisms consisted of the largest highly expressed DEG group [36]. This result seemed to be inconsistent with the phenotype of *dmc*, so we investigated energy metabolism in this study. Our results here showed both the chlorophyll content and net photosynthetic rate were significantly lower in *dmc* during tillering (Figure 5). The soluble sugar contents in tiller nodes of *dmc* were significantly less at the beginning of tiller. When the plants grew up, the photosynthesis capacity was significantly lower, and no sufficient carbohydrate could be supplied, leading to significantly decreased sugar contents in *dmc* (Figure 7).

The circadian rhythm—plant (ko04712) miRNA was one of the most enriched pathways. CRSP (CO_2_ Response Secreted Protease) is the target gene of miR9661-5P, which cleaves the pro-peptide EPF2 (Epidermal Patterning Factor 2), in turn, repressing stomatal development [39]. *TaCRSP* was lowly expressed in *dmc*. The Gs was low, but Ci was high in *dmc* compared to WT; the relationship among the three parameters in *dmc* requires further study.

The miRNAs miR-164, miR9664-3P, novel-393, novel-4827, novel-7652, novel-22869, novel-24656, novel-24657, novel-28763, novel-31210, novel-33200, novel-33201, novel-42734, and novel-44801 regulated carbohydrate metabolic pathway. Among them, the three novel DEMs—novel-22869, novel-393, and novel-4827—and their target DEG, glycerol-3-phosphate transporter 1 (G3P), were most significantly differentially expressed in *dmc* and WT; they were clearly related to carbohydrate metabolism, and G3P directly connected with the Cavin cycle of photosynthesis (Figure 6). 

Glycerol was reported to have important implications via glycerol-3-phosphate in not only lipid and carbohydrate metabolisms but also regulation of cellular energy homeostasis [40]. The circadian clock plays an important role in carbon partitioning and allocation [41]. Sugar sensing and signaling are involved in the control of growth and development during the entire plant life cycle [42]. High sugar accumulation during early seedling development may reflect undesirable growth conditions at a crucial developmental period [43], resulting in a reversible developmental arrest that acts as a protection mechanism. However, low sugar content can also inhibit normal development in plants. Several studies demonstrated that the targets of some miRNAs were involved in the metabolisms of carbon, sucrose, starch, etc. For example, overexpression of microRNA408 enhances photosynthesis, growth, and seed yield in diverse plants [44]. Rice overexpressing osa-miR171b extended vegetative growth and enhanced chlorophyll accumulation in leaves, and their tillers were thicker [25]. The glucose-induced repression of miR156 is dependent on the signaling activity of HEXOKINASE1. The defoliation-induced increase in miR156 levels can be suppressed by exogenous glucose [45]. Two miRNAs—miR394 and miR399—targeted APL2 and three sugar and carbohydrate metabolism-related genes (sugar transporter, invertase, and carbohydrate transmembrane transporter) [46]. Rice miR164b modulates the expression of *OsNAC2* and affects its stem development [27]. Sugars are not only energy materials for plant development but are also signaling molecules regulating plant growth and development. In this study, we identified a group miRNAs regulating photosynthetic systems and carbohydrate metabolism during wheat tillering; however, the exquisite regulatory networks of miRNA–mRNA–sugar need to be studied further.

### 3.4. The miRNAs Regulate Hormone Metabolism and Signaling in dmc

The rice *nal2/3* double recessive mutant encodes OsWOX3A, which acts as a positive regulator of *ARF1* and *ARF4* in leaves and is involved in the formation of tillers. OsWOX3A regulates the transcription of genes involved in auxin synthesis, signaling, and/or polar transport for lateral cell proliferation during vegetative [6]. The contents of IAA and ZA were less, but GA was more in *dmc* tiller primordia (Figure 8B). Accordingly, we speculated that some important miRNAs’ target genes were involved in plant hormone signal transduction pathway. A total of twelve DEMs were found in IAA and GA metabolism in this study (Figure 8A). *20_OX_2* was down-regulated by tae-miR164 in *dmc,* and *ARF11* was found targeted by up-regulated miRNAs novel-23239, novel-24049, novel-24662, novel-25328, and novel-1218. ARF proteins have been postulated to have important functions in plant developmental processes through the control of auxin signaling [47]. In *A. thaliana*, miR160-regulates *ARF10* and is involved in cell differentiation and proliferation [48]. *OsmiR396d* affects gibberellin and brassinosteroid signaling to regulate plant architecture in rice [49]. The miR393-TIR1/AFB2/AFB3 regulatory module was discovered to have multiple functions that manipulate the auxin response [50]. In *Arabidopsis*, auxin can induce the expression of the GA synthetic gene *GA20* [51]. However, cytokinins can inhibit the expressions of *GA20_OX_* and *GA3_OX_* [52]. Our results demonstrated that the miRNAs regulated hormone metabolism and signaling in *dmc* by regulating the expression of *20_OX_2*, and *ARF11,* etc. (Figure 8A; Table S9). In the end, the homeostasis of auxin, zeatin, and gibberellin was changed. The decrease in IAA and ZA content decreased cell proliferation and growth, whilst more gibberellin increased maturation. 

### 3.5. The miRNAs Regulate GRF in dmc

The most differentially expressed miRNAs in the regulation network were novel. They formed 102 pairs of miRNA–mRNA interactions. One third of the novel miRNAs—including novel-1218, novel-23239, novel-24049, novel-24656, novel-24657, novel-24662, novel-25328, novel-33200, novel-33201, and novel-44801—belong to the miR396 family, which regulate *GRF1*, *GRF2*, *GRF5*, *GRF* 9, *GRF10,* and *GRF12*, and formed 60 pairs of miRNA–mRNA interactions. The miR396/*GRF* regulatory module plays an important role in plant growth, signal transduction, and the stress response [53,54,55,56,57,58]. Recent studies have shown that *GRF* might be related to the decrease in tillering in low-tillering wheat [59]. Similarly, the miR396/*GRF* regulatory module played an important role in *dmc* at tillering stage.

### 3.6. A miRNA–mRNA Regulatory Network in dmc

**Figure 12:**
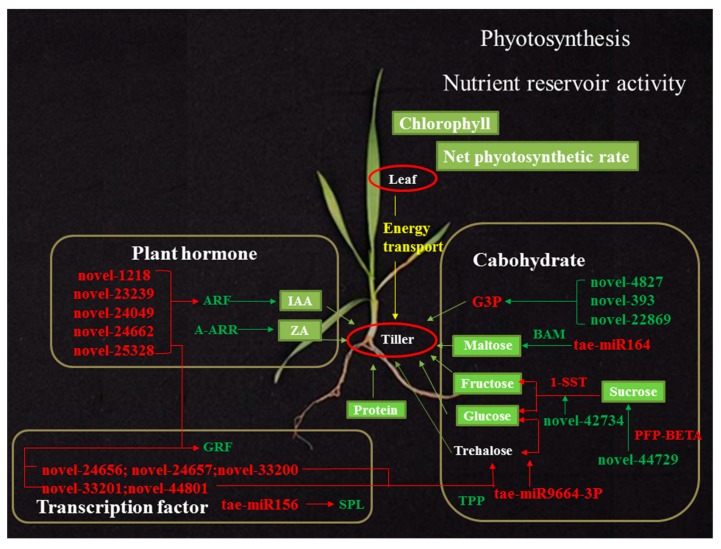
A molecular regulatory hypothesis of the *dmc*. Red: up-regulated; green: down-regulated compared to WT. Lower photosynthesis from the leaves produces fewer energy substances for transport to tiller nodes (Figure 5). Differentially expressed miRNAs regulated their target genes in the carbohydrate metabolism pathway and resulted in lower sugar content in tiller nodes (Figure 6 and Figure 7). Differentially expressed miRNAs also regulate transcription factor genes such as *SPL*, *GRF*, and plant hormone metabolism genes, such as *ARF* and *A-ARR* (Figure 8). Transcription factors, in turn, regulate other metabolisms such as carbohydrate and plant hormone metabolism pathways. In the end, the key substances that guarantee normal wheat tillering are in significant deficit, such as plant hormones IAA and ZA, soluble sugars, and proteins. These are the major factors restraining tillering in *dmc*.

In summary, we put forward a molecular regulatory hypothesis in *dmc* (Figure 12). Carbohydrate is transported from photosynthesizing source leaves to tiller and root. We found a lack of coordination, with increased carbohydrate needs but lower photosynthesis in the early tillering stage. The significant DEMs targeting a number of DEGs played an important role in regulating the carbohydrate metabolic pathway and resulted in a sugar-limited condition in *dmc* which downregulates biosynthetic activity to conserve energy and protect cells against nutrient stress while upregulating starch and protein degradation to sustain respiration and metabolic activity. Furthermore, the endogenous hormones IAA and ZA were decreased by a number of regulatory DEMs and DEGs. *GRF* and *SPL* are down-regulated by a number of miRNAs from the miR396 and miR156 families, respectively. In brief, the most important factors defining the no tillering and dwarfing phenotype of *dmc* were predicted to be some key miRNAs related to abnormal energy and hormone metabolisms (Figure 12).

## 4. Materials and Methods

### 4.1. Plant Materials and Growth Conditions

The wild type ‘Guomai 301′ has medium tillers but a high percentage of earbearing tillers. Mutant *dmc* with plant architecture variation was obtained from ethylmethame sulfonate (EMS) treated wheat cultivar Guomai 301. Both ‘Guomai 301′ and *dmc* were planted in the Experimental Farm of Henan Agricultural University, Zhengzhou, Henan Province, China (34°51′N, 113°35′E, 95 m asl) from 2016 [36]. All the experiments were designed as a complete random block. The WT and *dmc* were sown in plots of 3.0 m in length, and 2.0 m in width, the distance between rows was 0.25 m, and 20 seeds were planted in each row [60].

### 4.2. Morphological Observation 

The tiller samples of the mutant *dmc* and WT were prepared at five time points from the beginning to the end of tillering as described by He et al. [36]. The samples were observed with an inverted microscope (SRL-7045A, Beijing century science letter Scientific Instruments Co., Ltd., Beijing, China) and a scanner (MRS-9600TFU2L, China). All the images were captured by a camera (Nikon Coolpix 4500). Wheat developmental stages were described according to Zadoks [61].

### 4.3. RNA Preparation, small RNA Library Construction, and Sequencing

Two tiller bulk samples of *dmc* (S1, S2, and S3) and WT (S4, S5, and S6) were prepared at the three-leaf stage to four-leaf stage with three biological replicates. Each bulk sample included more than ten independent individuals. The total RNAs were extracted using Trizol reagent (TransGen Biotech, Beijing, China) according to the manufacturer’s protocol [36]. Each of the six miRNA libraries was constructed with 1.5 µg bulked total RNA using Small RNA Sample Pre Kit (NEB, Ipswich, MA, USA) following the manufacturer’s recommendations. T4 RNA ligase 1 and T4 RNA ligase 2 (truncated) were used to ligate adapters to the 3′ and 5ʹ terminals of the small RNAs. Then, the ligated small RNAs were reversely transcribed and amplified. Then the purified PCR products were sequenced on Illumina HiSeq2500 platform in BioMark Company (Beijing, China).

### 4.4. Analysis of the miRNA Data

The small RNA data were obtained by removing the low-quality reads and adapters from the raw data. The reads were trimmed and cleaned by removing the sequences smaller than 18 nt or longer than 30 nt. At the same time, Q30 of the clean data were also calculated. Then, the ribosomal RNA (rRNA), transfer RNA (tRNA), small nuclear RNA (snRNA), small nucleolar RNA (snoRNA), and other non-coding RNA (ncRNA) were filtered from the clean reads using the Rfam 11.0 (ftp://ftp.sanger.ac.uk/pub/databases/Rfam). The repeat sequences were filtered from the clean reads using the Repbase (http://www.girinst.org/). The remaining reads were used to identify known miRNAs and predict novel miRNAs by comparing with known miRNAs from miRBase 20 (http://www.mirbase.org). The clean reads were mapped to the reference genome (ftp://ftp.ensemblgenomes.org/pub/plants/release-32/fasta/triticum_aestivum/) using Tophat2 tool softs [62]. 

### 4.5. Analysis of the DEMs

The miRNA expression levels were normalized according to the expression of transcripts per million (TPM) [63]. The DESeq2 package in the statistics software R [64] was used to analyze DEMs between *dmc* and WT. DEMs between two samples were selected by setting the parameters of the fold change (|log_2_FC|) ≥ 1 and false discovery rate (FDR) ≤ 0.05.

### 4.6. MiRNA Target Gene Prediction, Interaction, and Functional Analysis

The targets of miRNAs were predicted using TargetFinder prediction software (http://www.bioinformatics.org/mirfinder) [65]. The predicted target genes of the DEMs were annotated referring to the databases Nr, Pfam, COG, Swiss-Prot, KO (KEGG Ortholog database), GO. Pathway and GO enrichment analyses were performed for target genes and target DEGs. The interaction networks between DEMs and their target DEGs were constructed using Cytoscape software [66].

### 4.7. Histochemical Observation

The small tillers of WT and mutant *dmc* were fixed in FAA solution (5 mL of formalin, 5 mL of acetic acid, and 90 mL of 70% ethyl alcohol). The samples were dehydrated, embedded in paraffin and sectioned with a rotary microtome as described by Geng et al. [67]. The distributions of starch and proteins were observed after the tissues were stained with Periodic Acid-Schiff 185 (PAS) and Naphthol Yellow S (NYS) (G1068, Servicebio). Photos were taken with a camera (Nikon Eclipse E100, Japan) and analyzed with CaseViewer software.

### 4.8. Determination of Chlorophyll Pigments and Photosynthesis

The chlorophyll mixture was extracted from fresh leaves (0.2 g) with three replicates using 95% alcohol at room temperature until the tissue was completely bleached. The Chl a, b, and ‘a + b’ were quantified spectrophotometrically at 470, 649, and 645 nm. Photosynthetic parameters of leaves were measured in the field from 9:00 to 11:00 a.m. using a Li-6400 portable photosynthesis system (Li-6400; LI-CORInc., Lincoln, NE, USA). The youngest fully expanded main-stem leaf was placed in light intensity of 1000 μmol m^−2^s^−1^, and the ambient CO_2_ concentration was approximately 400 μmol CO_2_ mol^−1^ air. All the experiments were repeated for three times.

### 4.9. Determination of Carbohydrate Contents 

The leaves and tiller nodes of mutant *dmc* and WT at three time points of the three-leaf stage (T_1_), over-winter stage (T_2_), and between the rising to jointing stage (T_3_) were sampled. The sugars were extracted and quantified according to the protocol described by Wang et al. [68]. The sugars of each sample were extracted from 100 mg of freeze-dried meal. 700 μL 70% alcohol was added to each sample, heated in a water bath at 70 °C for 2 h, then the same amount ultrapure water was added, mixed by vortex, and centrifuged at 13,200 g for 10 min. The supernatant was purified with chloroform three times, and the re-collected supernatant was used for quantification of the soluble sugars.

Four kinds of soluble sugars—glucose, sucrose, fructose, and maltose—were quantitatively determined by high-performance anion-exchange chromatography (HPAEC, ICS 5000, Dionex) equipped with a Carbopac™ PA-20 column (3 × 150 mm, Dionex) and a guard PA-20 column (3 × 30 mm, Dionex) with a pulsed amperometric detection (PAD). The mobile phase composed of sodium hydroxide and water (10:90, V/V) at a flow rate of 0.5 mL/min.

### 4.10. Determination of Hormone Contents

The hormones of each tiller node sample were extracted from 150 mg freeze-dried meal in 1 mL mixed solution (carbinol:0.5% acetic acid = 80:20) at 4 °C overnight, centrifuged at 8000 g for 10 min, and nitrogen was blown at 40 °C until no organic phase remained. The sample was decolored by adding 0.5 mL petroleum ether at 60–90 °C three times, and the supernatant petroleum ether was discarded. The pH value was adjusted to 2.8 by adding 0.1 mol/L citric acid, then extracted with acetic ether three times. The supernatant organic phases were combined and blown dry with a nitrogen blower. The sample was readied for testing by adding 0.5 mL of the mobile phase solution and filtered with a needle filter.

IAA and ZA were determined by a high performance liquid chromatography (HPLC) (Agilent 1100) system with a Kromasil C18 (250 mm × 4.6 mm, 5 μm) reversion phase chromatography column at 35 °C. The mobile phase composed of carbinol and 1% acetic acid solution (4: 6, V/V) at a flow rate of 0.8 mL/min, and the UV absorbance was monitored at 254 nm. GA3 was determined by HPLC (Rigol L3000) system with an Kromasil C18 (250 mm × 4.6 mm, 5 μm) reversion phase chromatography column at 30 °C. The mobile phase composed of carbinol and 1% phosphate solution (35: 65, V/V) at a flow rate of 1 mL/min, and the UV absorbance was monitored at 210 nm.

### 4.11. Real-time qRT-PCR

Total RNA of the tiller primordia of mutant *dmc* and Guomai 301 at three-leaf stage to four-leaf stage were extracted using Trizol reagent (TransGen Biotech, Beijing, China). For the expression patterns of DEMs, reverse transcription was carried out using TransScript^®^ miRNA First-Strand cDNA Synthesis SuperMix (TransGen Biotech, Beijing, China). The qPCR was performed using TransStart^®^ Tip Green qPCR SuperMix (TransGen Biotech, Beijing, China). For the target genes, reverse transcription was performed using TransScript^®^ All-in-One First-Strand cDNA Synthesis SuperMix for qPCR (TransGen Biotech, Beijing, China). Real-time qRT-PCR was performed using TransStart^®^ Top Green Qpcr SuperMix (2×) (TransGen Biotech, Beijing, China) according to the manufacturer’s protocol on the CFX ConnectTM Real-Time System (Bio-Rad, Hercules, CA, USA). The data were normalized by comparing to the expression of *U6* for miRNAs and to the wheat *actin* gene for the target genes based on calculations of 2^−ΔΔCT^. All the primer sequences of miRNAs/target genes were listed in Table S12.

## 5. Conclusions

We identified a set of miRNAs from *dmc* and WT and established a miRNA–mRNA regulatory network for tiller development in *dmc*. There are 91 DEMs between *dmc* and WT; 40 significant DEMs played important roles during wheat tillering. Many DEMs belong to the miR396 family and regulate the expression of the key developmental regulation genes, *ARF11*, *GRF1*, *GRF2*, *GRF5*, *GRF9*, *GRF10,* and *GRF12*. There are 26 important DEMs regulating carbohydrate and hormone metabolisms. The miRNAs novel-22869, novel-393, and novel-4827—involved in carbohydrate metabolisms—and novel-23239, novel-24049, novel-24662, novel-25328, novel-1218—involved in hormone metabolisms—are supposed to be essential regulators for tillering in *dmc*. Many other biological functions are likely being modified by decreased photosynthetic capabilities. The lower photosynthesis, insufficient energy, and abnormal hormone homeostasis are the major factors which restrict tillering in wheat mutant *dmc*.

## Figures and Tables

**Table 1:** Major metabolic pathways related to wheat tillering (*P* < 0.05).

Kegg Pathway	Ko Id	*P* Value	Corrected *P* Value
Protein processing in endoplasmic reticulum	ko04141	6.01 × 10^−10^	3.54 × 10^−8^
Spliceosome	ko03040	1.20 × 10^−5^	7.05 × 10^−4^
Circadian rhythm—plant	ko04712	3.20 × 10^−4^	1.89 × 10^−2^
Degradation of aromatic compounds	ko01220	1.01 × 10^−2^	5.97 × 10^−1^
Plant-pathogen interaction	ko04626	1.17 × 10^−2^	6.88 × 10^−1^
Homologous recombination	ko03440	1.61 × 10^−2^	9.50 × 10^−1^
Fructose and mannose metabolism	ko00051	1.69 × 10^−2^	1.00 × 10^0^
Butanoate metabolism	ko00650	2.52 × 10^−2^	1.00 × 10^0^
Arginine and proline metabolism	ko00330	3.04 × 10^−2^	1.00 × 10^0^

## Supplementary Materials

Supplementary Materials can be found at https://www.mdpi.com/1422-0067/20/18/4586/s1.
