# Supplementary figures and images for: The miRNA–mRNA Networks Involving Abnormal Energy and Hormone Metabolisms Restrict Tillering in a Wheat Mutant dmc
Source: Int J Mol Sci. 2019 Sep 17;20(18):4586. doi: 10.3390/ijms20184586 (PMC6770018; doi:10.3390/ijms20184586)

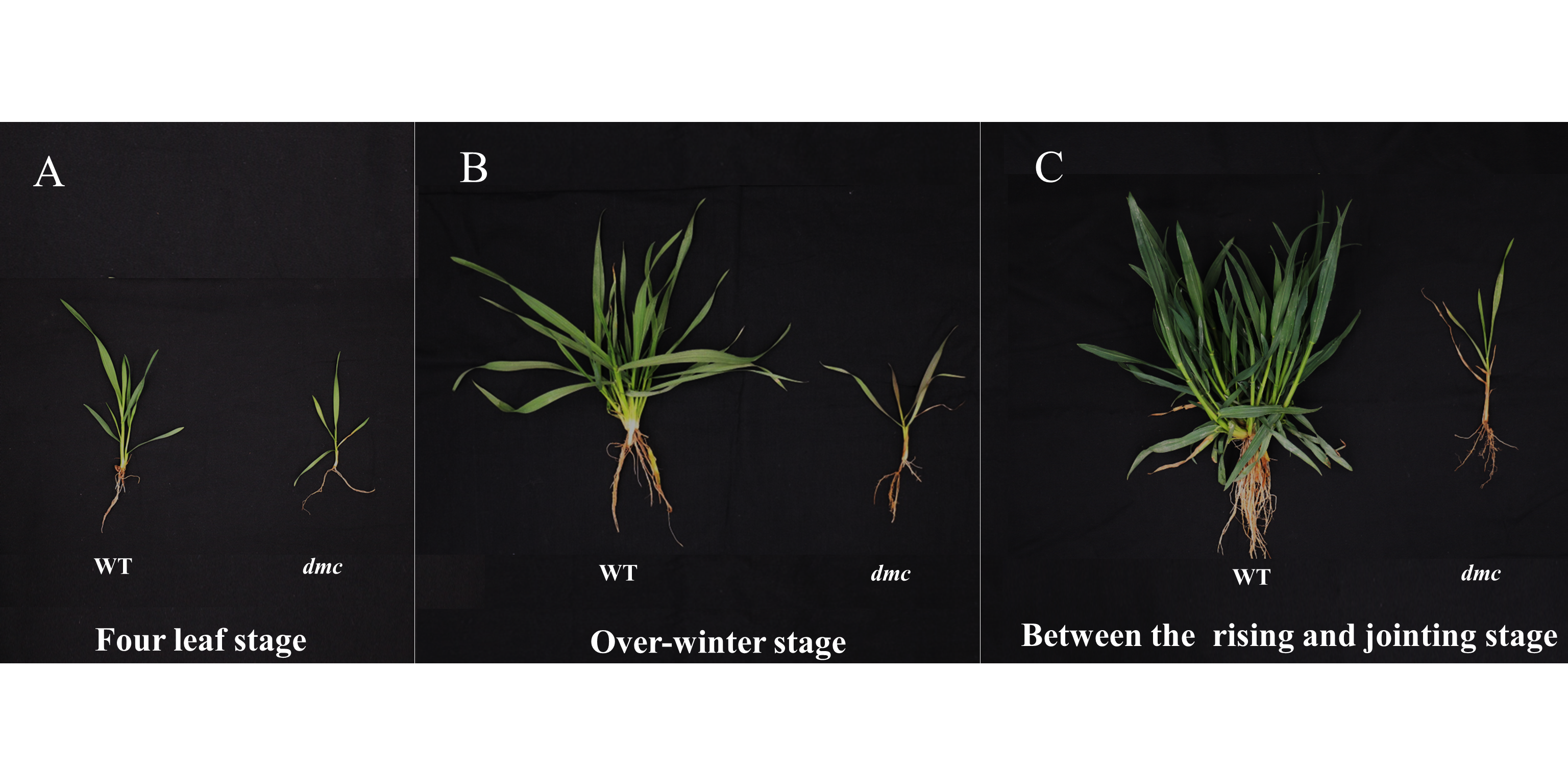

Supplement: Supplementary file 1 [file ijms-20-04586-s001.zip › Supplementary Files/Figure S1.tif]

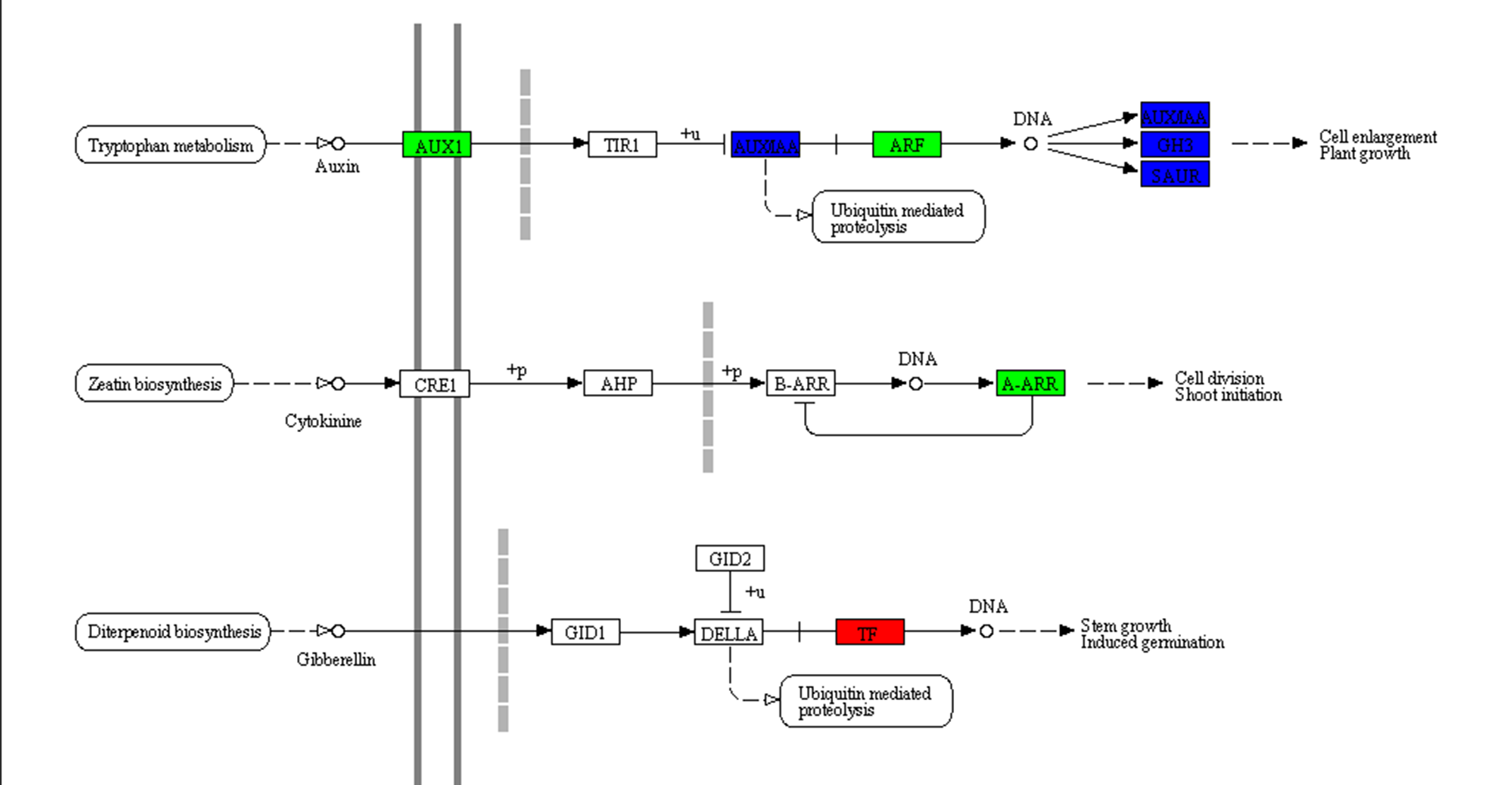

Supplement: Supplementary file 1 [file ijms-20-04586-s001.zip › Supplementary Files/Figure S3.tif]

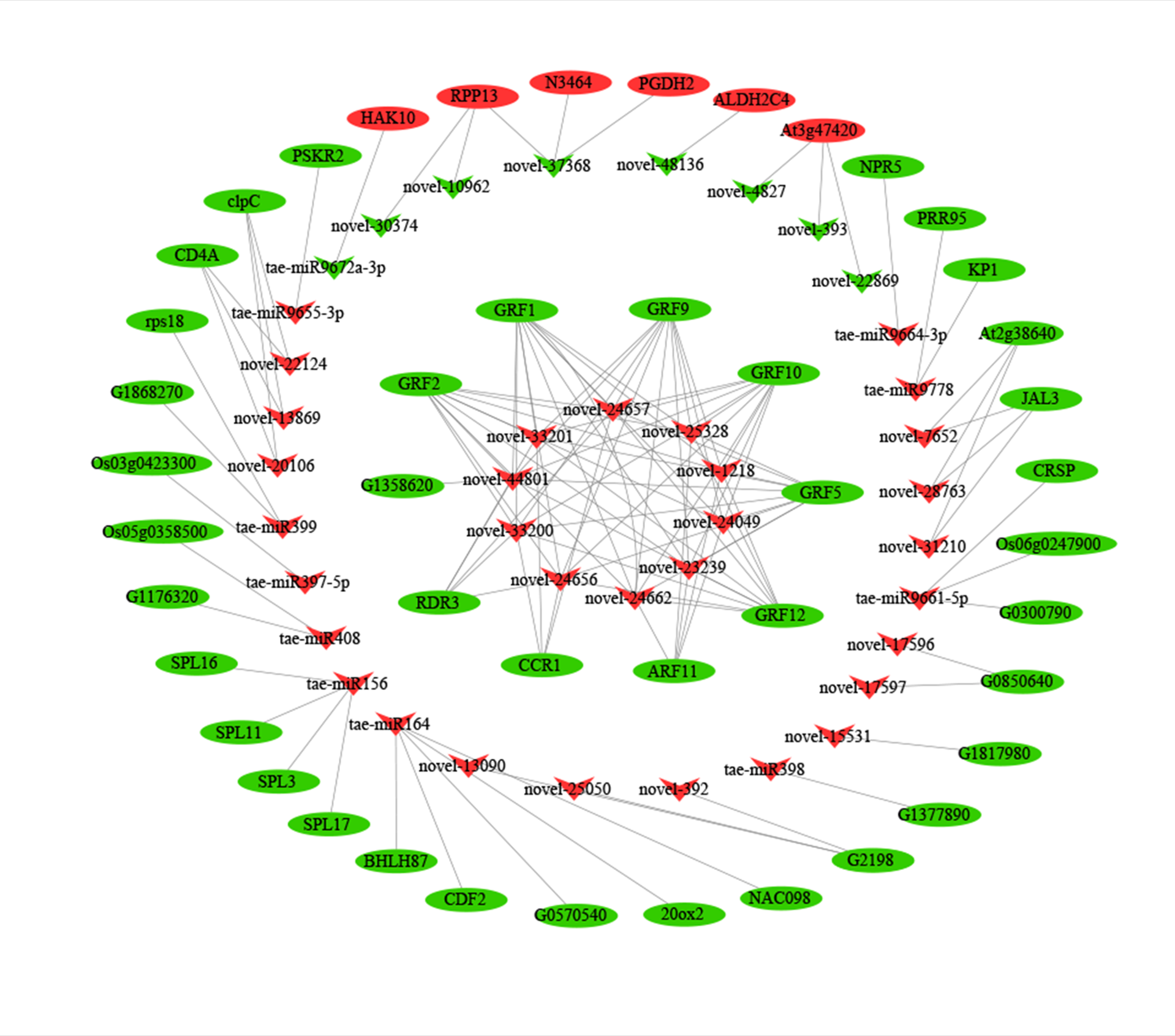

Supplement: Supplementary file 1 [file ijms-20-04586-s001.zip › Supplementary Files/Figure S4.tif]
